# Understanding polycystic ovary syndrome from the patient perspective: a concept elicitation patient interview study

**DOI:** 10.1186/s12955-017-0736-3

**Published:** 2017-08-18

**Authors:** Mona L. Martin, Katarina Halling, Daniel Eek, Meaghan Krohe, Jean Paty

**Affiliations:** 1 Health Research Associates, 6505 216th St SW, Mountlake Terrace, Seattle, WA 98043 USA; 2 AstraZeneca Gothenburg, Pepparedsleden 1, 431 83 Mölndal, SE Sweden; 3 Adelphi Values, 7th Floor, 290 Congress St, Boston, MA 02210 USA; 4 Quintiles, 8 Skyline Dr, Hawthorne, New York, NY 10532 USA

**Keywords:** Concept elicitation, Impacts, Patient perspective, Polycystic ovary syndrome, Qualitative interviews, Symptoms, Unmet need

## Abstract

**Background:**

The aim of this study was to explore the need for a new disease-specific patient reported outcome (PRO) measure for use in clinical trials of drugs designed to target the underlying causes of polycystic ovary syndrome (PCOS), and in the process contribute to our understanding of the symptoms and impacts that define the patient experience with PCOS.

**Methods:**

Semi-structured interviews were conducted in 20 women diagnosed with PCOS according to the Rotterdam criteria who had not menstruated in the previous month. The relative importance of PCOS symptoms and impact concepts to patients was determined by analyzing the frequency of their expression in the interview transcripts. These insights were compared to clinicians’ perceptions of PCOS.

**Results:**

Pain- and discomfort-related symptoms accounted for the highest proportion (27.6%) of the 735 patient expressions, although clinicians did not consider pain to be important to patients with PCOS. The most frequently expressed individual symptoms were cramping (70% of patients; 14.7% of concepts), irregular menstruation (95% of patients; 12.2% of concepts), facial hair growth (75% of patients; 10.6% of concepts), heavy bleeding (70% of patients; 8.8% of concepts), infertility (70% of patients; 5.4% of concepts), and bloating (60% of patients; 5.2% of concepts). Cramping, heavy bleeding, and bloating were not identified by clinicians as being important to patients with PCOS. The impacts most frequently reported by patients with PCOS related to emotional well-being (e.g. anxiety/stress) and coping behaviors (e.g. acne medication, hair removal).

**Conclusions:**

The only validated PCOS-specific PRO, the PCOSQ, does not capture some key PCOS symptoms and impacts expressed by patients with PCOS, most notably those related to pain and discomfort, bleeding intensity and coping behaviours. Furthermore, some key PCOS symptoms may be under-recognized in the clinic.

**Electronic supplementary material:**

The online version of this article (doi:10.1186/s12955-017-0736-3) contains supplementary material, which is available to authorized users.

## Background

Polycystic ovary syndrome (PCOS) is one of the most common endocrine disorders in women, with prevalence estimates of 4–8% reported among those of reproductive age [1–4], although underdiagnosis means that the true prevalence may be much higher [5]. The exact causes of PCOS are unknown, but it is thought to be a result of hormonal disturbances (increased androgens and/or insulin) induced by a combination of genetic and environmental factors (e.g. lifestyle/obesity) [6, 7].

Qualitative interviews in women with PCOS have identified a range of symptoms that define their experience with this disease, including hirsutism, infertility, irregular menstruation, weight issues and acne [8–16]. Impacts of PCOS include reduced psychological and emotional well-being, negative self-image, and impaired physical, sexual, social and cognitive functioning [8–10, 12–15, 17]. Not surprisingly, PCOS is associated with significantly impaired quality of life and psychosocial well-being [18]. Treatment and management of PCOS is broadly based on its reproductive (hyperandrogenism, hirsutism, ovulatory and menstrual dysfunction, infertility), metabolic (increased type 2 diabetes and cardiovascular risks), and psychological (anxiety, depression, negative body image) consequences [19].

The most widely used disease-specific patient reported outcome (PRO) instrument available for PCOS is the Polycystic Ovary Syndrome Health-Related Quality of Life Questionnaire (PCOSQ) [20], of which a modified version also exists [21], as well as several language-specific adaptations [22–25]. However, the PCOSQ has several limitations that may limit its use in clinical trials. First, it was designed to measure health-related quality of life rather than to specifically address symptoms and symptom-related impacts, which are more proximal to the condition and more interpretable from a drug regulatory standpoint. Second, the development of the PCOSQ as described in the literature leaves doubts as to its ability to fully reflect the concepts relevant to the patient experience, thus raising questions regarding its content validity, which is of utmost importance for PRO instrument development [26]. Finally, the ability of this instrument to detect treatment-related change varies in the literature and a minimal clinically important difference has not been established [27].

AZD4901 (now MLE4901) is a high-affinity antagonist of the human neurokinin-3 receptor that was hypothesized to target the central pathophysiology of PCOS by blocking luteinizing hormone hypersecretion. As part of the clinical development program for this drug, we conducted qualitative interviews with patients with PCOS and also with clinical PCOS experts. This work was initiated with the goal of developing a PCOS-specific PRO with demonstrable content-validity for use in clinical trials of AZD4901, in line with industry best-practice [28]. It also presented an opportunity to contribute to current knowledge regarding the symptoms and impacts of greatest relevance to patients with PCOS. Here we report on the results of the patient interviews, and what we learned relative to clinicians’ perceptions.

## Methods

### Patient interviews: Concept elicitation study

#### Study conduct

Three primary care treatment clinics specializing in women’s health in New Orleans, Louisiana; Hershey, Pennsylvania; and Spokane, Washington were enrolled as patient recruitment sites. Each site was asked to use standardized forms to identify, screen, recruit, confirm eligibility, and collect descriptive data for 5–10 patients. All participants provided written informed consent, and were scheduled for individual, face-to-face, qualitative interviews. Interviews were conducted by trained project staff with several years of experience in qualitative research. Semi-structured interview guides were used to elicit aspects of the patients’ experiences with PCOS. The study was reviewed and approved by Quorum Review, Seattle, WA, USA and the Institutional Review Board at Pennsylvania State University, PA, USA.

#### Patient sample

The current study was initiated as part of the clinical development program for AZD4901, a high-affinity antagonist of the human neurokinin-3 receptor that is hypothesized to target the central pathophysiology of PCOS by blocking luteinizing hormone hypersecretion. Patient selection criteria were thus necessarily aligned with those used in a recent phase 2a study of AZD4901 (ClinicalTrials.gov identifier: NCT01872078). Specifically, women aged 18–45 years with a body mass index of 18–40 kg/m^2^ (inclusive) and a clinical diagnosis of PCOS were recruited. A clinical diagnosis of PCOS was defined, based on the Rotterdam criteria [29], as the presence of all of the following: polycystic ovaries documented by ultrasound; free testosterone >85% of the upper limit of the reference range for females, as well as clinical signs of exuberant testosterone effects (acne and/or hirsutism); and amenorrhoea or oligomenorrhoea (defined as ≤6 menses per year). Patients with PCOS who had menstruated in the month prior to screening were excluded. This criterion was applied in the phase 2a study of AZD4901 because menstruation affects luteinizing hormone pulse characteristics, which was a key endpoint measurement. Individuals were also excluded if they were peri-menopausal or had reached natural menopause (defined as follicle-stimulating hormone >10 IU/L), had undergone a hysterectomy or bilateral oophorectomy, were pregnant or lactating, had a medical condition or disorder that could compromise their ability to give written informed consent and/or interfere with their ability to successfully participate in the study, or were involved in any aspect of the planning or conduct of the study. Individuals also needed to have a sufficient understanding of English to complete the questionnaires and take part in the open-ended interviews.

#### Concept elicitation

Two female interviewers were involved in conducting the qualitative interviews. Each interview lasted approximately 60 min and took place in a private room at the recruiting clinic. The interview guide (see Additional file 1) focused first on PCOS symptoms and then moved to issues related to the impacts that patients experienced as a result of those symptoms. The last section of the interview included questions about treatment and treatment goals.

The interview guide was designed to elicit concepts spontaneously in response to open-ended questions. Examples of open-ended questions included “Can you describe any physical sensations [symptoms] you had that were related to your PCOS?” and “What activities have you had to cut back on [impacts] because of your PCOS?”. For symptoms, concepts were also elicited using a day reconstruction exercise, which asked patients to describe a typical day with PCOS. Specifically, after being asked to select a recent day when they experienced PCOS symptoms, patients were asked questions such as “Can you describe the very first symptom you remember having right after you woke up that morning?” and “What other symptoms did you notice as you went through your morning routine?”

Once patients were sure they had brought up all the concepts they could think of in response to the open-ended questions, specific probes were used. These were first used to identify any other concepts that patients with PCOS recalled they may have experienced (“Now I’m going to describe some additional symptoms that some women with PCOS describe. As I read the list, please tell me if you recognize experiencing any of these.”). This section was included because most patients only provide their most troublesome or most frequent symptoms in response to open-ended questions – not everything they have experienced. Probing questions where then also used to obtain further details about the concepts already mentioned in response to open-ended or probing questions (e.g. “How long does this symptom usually last?”).

Near the end of the qualitative interview, worksheet exercises were used to attain numeric ratings of symptom severity (0 = none; 10 = extremely severe), symptom bother (0 = not bothersome at all; l = extremely bothersome), and the difficulty of dealing with impacts (impact difficulty; 0 = no difficulty at all; 10 = extremely difficult).

#### Qualitative analysis

All enrollment and demographic data were entered into SPSS for Windows (version 11.5, IBM, New York, USA) in order to generate descriptive tables. Word files of transcribed audio recordings from the patient interviews were loaded into ATLAS.ti (version 5.0; Scientific Software Development, Berlin, Germany) for concept coding. A coding framework was developed so that the coded symptom and impact concepts could be organized according to content. The preliminary coding framework evolved further as concepts were identified from the interview transcripts. Concepts were identified in the transcript text and tagged to an appropriate code stem (or, if necessary, a new code stem was created). For example, eight patient expressions were found in the transcript database that reported content about nausea. The patient language (e.g. “felt nauseous all the time”, “I did vomit”, “had major nausea”) were connected to a code stem for “nausea” so these expressions could be grouped together for qualitative evaluation.

Two randomly chosen transcripts (10% of the qualitative database) were dual coded to evaluate the degree of inter-rater agreement in the coding process. Saturation of concept was used to determine whether or not additional information was still forthcoming and whether the data set could be considered to be complete. Saturation of concept was evaluated by ordering the transcripts chronologically and then creating groups of four or five transcripts each. After concepts appearing in the first transcript group had been coded, each subsequent group was evaluated and compared to the previous group in order to identify the appearance of any new concept codes. Saturation of concept was considered to be met when no new concepts appeared.

### Clinician interviews

One-on-one semi-structured telephone interviews (approximately 60 min long) were conducted with five clinical PCOS experts, in line with regulatory requirements for the development of PRO instruments for use in drug trials. During the interviews, clinicians were asked to determine how relevant or important they thought PCOS symptom and impact concepts identified from the literature (Additional files 2 and 3) were to their patients, and whether any revisions or additions should be made. The clinical expert interviews were conducted before the patient interviews, and the results informed both the preliminary coding framework and the symptoms and impacts included in the follow-up probe sections of the interview guide.

## Results

### Patient interviews: Concept elicitation study

A total of 20 qualitative patient interviews were conducted. The characteristics of the study participants are presented in Table 1.

**Table 1:** Patient demographic and clinical characteristics

Characteristic	Patients (*N* = 20)
Age (years)	
Mean (SD)	29.2 (5.9)
Median (range)	30.4 (18.0 − 38.9)
Marital status, *n* (%)	
Married or living as married	14 (70)
Divorced	1 (5)
Never married	5 (25)
Highest level of education completed, *n* (%)	
Less than high school	1 (5)
High school	8 (40)
Some college	7 (35)
Bachelor’s degree	3 (15)
Graduate or professional school	1 (5)
Current employment status, *n* (%)	
Full-time	11 (55)
Part-time	3 (15)
Not employed	6 (30)
Ethnicity, *n* (%)	
White	19 (95)
Other: White and Hispanic	1 (5)
Time since PCOS diagnosis (years)	
Mean (SD)	6.3 (5.0)
Median	5.5
Range	0 − 15

*PCOS* Polycystic ovary syndrome, *SD* Standard deviation

#### Data quality assessments

Most (83%) of all symptom and impact concept codes appeared after the first group of five interviews. The remaining 17% of concepts appeared after the second and third interview groups, after which no new codes were identified. This demonstrated saturation of concept by the completion of the third group of interview transcripts. High inter-rater agreement was demonstrated between coders, with 88.6% and 91.1% agreement in terms of the total number of concepts identified, and 97.4% and 98.8% agreement in terms of the codes that were assigned to the concepts. An ideal target for agreement in code assignment is above 90%.

#### Qualitative analysis of symptoms

During the interview process, patients expressed a total of 735 symptom concepts (Table 2). The concept groups accounting for the highest proportion of patient-expressed symptoms were “Pain and Discomfort”, “Hair Loss and Growth”, “Menstruation”, and “Bleeding”. Within the concept groups, the most frequently coded symptoms were cramping, irregular menstruation, facial hair growth, and heavy bleeding. These were also the symptoms most frequently reported spontaneously. Frequently reported symptoms in the remaining concept groups included bloating, infertility, and acne. However, bloating and acne were generally only mentioned by patients after probing for experience with these symptoms.

**Table 2:** Symptom concept code frequencies by concept groups

Symptom concept groups and concepts, *n* (%)	Expressions (*N* = 735)	Contributing patient transcripts (*N* = 20)
Pain and Discomfort	203 (27.6)	–
Cramping	108 (14.7)	14 (70.0)
Bodily pain	40 (5.4)	8 (40.0)
Menstrual pain	19 (2.6)	5 (25.0)
General expressions of pain	18 (2.4)	6 (30.0)
Pain during sexual intercourse	11 (1.5)	5 (25.0)
Hot flashes	7 (1.0)	2 (10.0)
Hair Loss and Growth	119 (16.2)	–
Facial hair growth	78 (10.6)	15 (75.0)
Body hair growth	26 (3.5)	9 (45.0)
Hair loss	15 (2.0)	5 (25.0)
Menstruation	116 (15.8)	–
Irregular menstruation	90 (12.2)	19 (95.0)
No menstruation	26 (3.5)	11 (55.0)
Bleeding	100 (13.6)	–
Heavy bleeding	65 (8.8)	14 (70.0)
Bleeding of long duration	25 (3.4)	8 (40.0)
Light bleeding	10 (1.4)	5 (25.0)
Weight and Bloating	89 (12.1)	–
Bloating	38 (5.2)	12 (60.0)
Weight gain	18 (2.4)	10 (50.0)
Fluctuating weight	16 (2.2)	6 (30.0)
Difficulty losing weight	15 (2.0)	8 (40.0)
Other weight-related symptoms	2 (0.3)	1 (5.0)
Infertility and Anovulation	63 (8.6)	–
Infertility	40 (5.4)	14 (70.0)
No ovulation	23 (3.1)	7 (35.0)
Skin Changes	37 (5.0)	–
Acne	28 (3.8)	9 (45.0)
Darkened skin	8 (1.1)	2 (10.0)
Other skin-related symptoms	1 (0.1)	1 (5.0)
Additional Symptoms	8 (1.1)	–

**Fig. 1:**
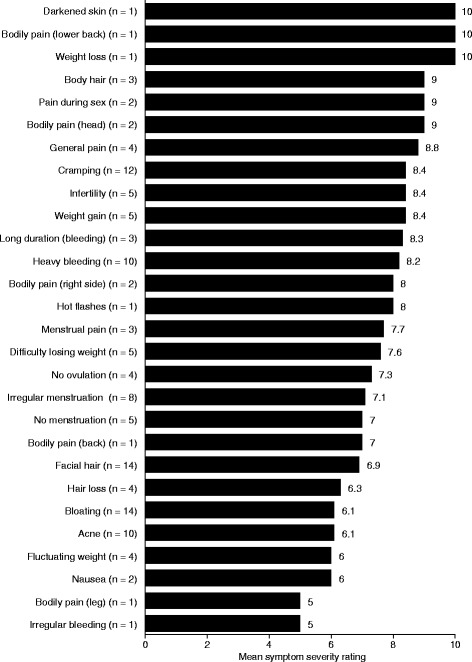
Patient PCOS symptom severity ratings. Patients were asked to provide ratings of symptom severity (“how bad is it when it’s at its worst”) using a numeric rating scale from 0 (none) to 10 (extremely severe). Note: Severity rating exercises were conducted during the detailed probes section of the interview. In contrast, coded frequency data (Tables 2 and 3) were based on analyses of all coded concepts in the entire transcript dataset. Because collection of these data occurred at different times, and one was patient-based while the other was based on the code itself as the unit of analysis, there will be instances where the number of transcripts contributing to code frequency data and the number of patients contributing to the severity rating data differ

Severity ratings given by patients for their symptoms are provided in Fig. 1. Of the symptoms rated for severity by at least five patients, the highest mean severity scores (8.2–8.4) were for cramping, infertility, weight gain, and heavy bleeding, followed by difficulty losing weight (7.6), irregular menstruation (7.1), no menstruation (7.0), facial hair growth (6.9), bloating, and acne (both 6.1). Several symptoms (darkened skin, pain in the lower back, and weight loss) received a maximum severity score of 10 but were rated by only one patient.

**Fig. 2:**
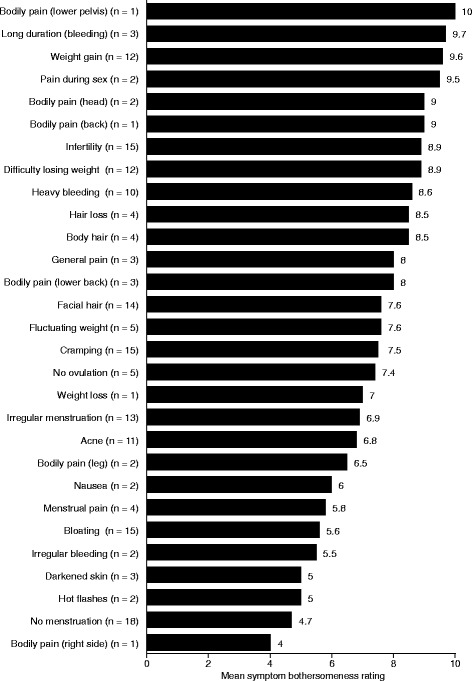
Patient PCOS symptom bother ratings. Patients were asked to rate how much each symptom they experience bothers them, using a numeric rating scale from 0 (not bothersome at all) to 10 (extremely bothersome). Note: Bother rating exercises were conducted during the detailed probes section of the interview. In contrast, coded frequency data (Tables 2 and 3) were based on analyses of all coded concepts in the entire transcript dataset. Because collection of these data occurred at different times, and one was patient-based while the other was based on the code itself as the unit of analysis, there will be instances where the number of transcripts contributing to code frequency data and the number of patients contributing to the bother rating data differ

Symptom bother ratings provided by patients are reported in Fig. 2. The highest mean bother score for symptoms rated by at least five patients was given for weight gain (9.6). This was followed by infertility, difficulty losing weight, heavy bleeding (8.6–8.9), facial hair growth, fluctuating weight, cramping, no ovulation (7.4–7.6), irregular menstruation (6.9), acne (6.8), bloating (5.6), and no menstruation (4.7).

#### Qualitative analysis of impacts

A total of 549 patient-expressed impact concepts were coded across the 20 interview transcripts (Table 3). The concept groups reported with greater predominance were “Emotional Impacts”, “Coping Behaviors”, “Sleep and Energy Restrictions”, and “Social/Lifestyle Limitations and Restrictions”. Within these concept groups, the most frequently coded impacts were anxiety/stress, the use of medication, tiredness, and impaired relationships.

**Table 3:** Impact concept code frequencies by concept group

Impact concept groups and concepts, *n* (%)	Expressions (*N* = 549)	Contributing patient transcripts (*N* = 20)
Emotional Impacts	185 (33.7)	–
Anxiety/stress	34 (6.2)	11 (55.0)
Frustration	26 (4.7)	8 (40.0)
Embarrassment	20 (3.6)	8 (40.0)
Worry and concern	19 (3.5)	10 (50.0)
Self-image affected	19 (3.5)	6 (30.0)
Mood swings	19 (3.5)	5 (25.0)
Irritability	16 (2.9)	7 (35.0)
Depression	12 (2.2)	8 (40.0)
Jealousy	6 (1.1)	3 (15.0)
Low self-esteem	6 (1.1)	3 (15.0)
Self-blame	5 (0.9)	3 (15.0)
Other emotional difficulties	3 (0.5)	2 (10.0)
Coping Behaviors	173 (31.5)	–
Use of medications	55 (10.0)	16 (80.0)
Dietary changes	31 (5.6)	12 (60.0)
Resting and relaxing	19 (3.5)	10 (50.0)
Shaving	16 (2.9)	7 (35.0)
Trimming	15 (2.7)	6 (30.0)
Increased exercise	10 (1.8)	6 (30.0)
Other coping behaviors	8 (1.5)	5 (25.0)
Use of heat/heating pad	6 (1.1)	4 (20.0)
Waxing	6 (1.1)	4 (20.0)
Plucking	4 (0.7)	3 (15.0)
Using clothing as coverage	3 (0.5)	2 (10.0)
Sleep and Energy Restrictions	80 (14.6)	–
Tiredness	29 (5.3)	8 (40.0)
Decreased energy	11 (2.0)	6 (30.0)
Difficulty staying asleep	10 (1.8)	4 (20.0)
Impaired sleep quality	10 (1.8)	4 (20.0)
Lack of energy	9 (1.6)	5 (25.0)
Sleep apnea	6 (1.1)	3 (15.0)
Difficulty falling asleep	5 (0.9)	4 (20.0)
Social/Lifestyle Limitations and Restrictions	65 (11.8)	–
Relationships impaired	34 (6.2)	11 (55.0)
Sexual activity affected	17 (3.1)	9 (45.0)
Social activity limitations	8 (1.5)	4 (20.0)
Limitations to leisure activities	6 (1.1)	3 (15.0)
Difficulty Doing Daily Activities	44 (8.0)	–
General difficulty with daily activities (20)	3.6 (6)	6 (30.0)
Difficulty with professional responsibilities (13)	2.4 (5)	25.0)5 (25.0)
Difficulty with exercise (7)	1.3 (4)	20.0)4 (20.0)
Difficulty with household responsibilities (4)	0.7 (3)	15.0)3 (15.0)
Additional Impacts	2 (0.4)	2 (10.0)

*PCOS* Polycystic ovary syndrome

Of impacts rated by at least five patients, mean scores of 8.0–8.6 were given for difficulties dealing with shaving (*n* = 5), embarrassment (*n* = 5), and impacts on sex (*n* = 10) and leisure (*n* = 5). The next highest scores (7.0–7.6) were for difficulties dealing with mood swings (*n* = 5), frustration (*n* = 5), and impaired relationships (*n* = 8). These were followed by worry (*n* = 14), anxiety/stress (*n* = 5), impaired exercise (*n* = 6), and being irritable (*n* = 5) or tired (*n* = 9), which had mean scores of 6.1–6.9, and depression (*n* = 6) with a mean score of 5.0.

#### Qualitative analysis of treatment effectiveness

At the close of interviews, patients were asked about their experience with treatment for PCOS. Negative factors leading to treatment dissatisfaction were mostly related to adverse effects of the medication, including nausea and vomiting, weight gain, heavy bleeding during menstruation, mood changes, diarrhea, cramps, and headaches. Lack of treatment responsiveness (i.e. inability to get pregnant, facial hair, anovulation) was also cited as a reason for dissatisfaction. Treatment success was described as being fertile and having regular menstrual cycles, and eliminating facial hair, cramps, ovarian cysts, and bloating.

### Clinician interviews: Overlap with patient PCOS experience

Bodily Pain was not considered by clinicians to be relevant or important to patients with PCOS. In contrast, the concept group Pain and Discomfort accounted for the highest proportion of symptoms expressed by patients with PCOS during their interviews (Table 2). The most frequent symptom expressed by patients in relation to pain was cramping, which was not always associated with menstruation (e.g. “I'm having cramping without a period”). Cramping was not identified by clinical experts as being important or relevant to patients with PCOS, but was the symptom most frequently expressed by patients. This is somewhat surprising given the intensity of the language patients often used to describe cramping. For example, one patient said “I just remember being kinda curled up in a fetal position, rocking you know, thinking it's gotta go away tomorrow”, while another said “the pain is excruciating, like someone is poking you with a knife, like someone stabbed you”.

Issues relating to menstruation (irregular periods/no periods) were identified by the clinical experts as being of relevance and importance to patients with PCOS. However, heavy bleeding and bleeding of long duration, which did not come up during interviews with clinicians, were among the symptom concepts most frequently expressed by patients. The relevance of heavy bleeding to patients with PCOS appeared to be mainly related to the inconvenience that it caused (e.g. “whenever it is excessive, have to change the largest scale tampon less than in an hour”) and, perhaps more importantly, fear and worry over the perceived health consequences (e.g. “I would bleed so much, fear was always ‘am I hemorrhaging?’”).

The clinical experts identified sleep apnea as an important impact of PCOS, but this impact was not often expressed by patients during their interviews. However, patients did report generally disturbed sleep patterns (e.g. “when I fall asleep, I wake up every 3 hours”) in relation to a variety of factors, including cramping, heavy bleeding, migraine, and panic attacks, and also reported reduced energy levels (e.g. “my period makes me feel tired”).

Compensatory or coping behaviors (e.g. use of medication, diet changes, hair removal techniques) were identified from both the clinician and patient interviews as being of relevance and importance to patients with PCOS. Although bloating was generally only mentioned by patients after probing for this symptom, it was expressed at a sufficiently high frequency to be considered an important symptom of PCOS and was not mentioned by the clinical experts.

**Fig. 3:**
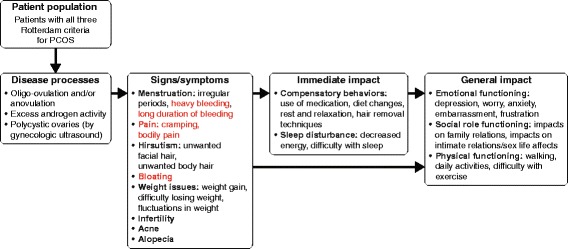
Disease model of the signs, symptoms, and impacts that are important and relevant to patients with PCOS based data combined from the patient interviews described above, and the interviews conducted with clinical PCOS experts. Red text indicates concepts that were identified from the patient interviews but which were not highlighted by clinicians as being relevant or importance to the patients experience with PCOS

The PCOS disease model presented in Fig. 3 shows the signs, symptoms, and impacts identified as being of relevance to patients with PCOS, on the basis of data combined from the patient interviews described above, and the interviews conducted with clinical PCOS experts.

## Discussion

In this study, we conducted qualitative interviews with patients who have PCOS in order to understand the symptoms and impacts they experience living with this common endocrine disorder. Some of the concepts frequently expressed by patients (and thus considered important from their perspective) were not identified by clinicians and/or are not captured by current PCOS-specific PRO instruments, highlighting potential gaps in the current knowledge and understanding of this syndrome.

Almost a quarter of all symptom concepts expressed by patients during their interviews were in relation to the pain and discomfort that they experience with PCOS. However, pain was unanimously viewed by the clinical PCOS experts interviewed as not being “relevant” or “important” to patients with PCOS. Other qualitative interview studies have identified pain in various forms as being part of the patient experience with PCOS, including bodily, abdominal, pelvic or belly pain [8, 10, 12, 15], sexual pain [8] and headaches [10]. The most widely used and only validated PCOS-specific PRO, the PCOSQ, incorporates only one pain-related question on headaches. Pain items are also not included in PCOSQ-50, which was recently developed based on interviews with Iranian women [30].

The most frequently reported pain- and discomfort-related symptom was cramping, which was also the most frequently expressed of all symptoms. Menstrual cramping is captured by the PCOSQ. However, an important feature of the nature of the cramping reported by women with PCOS is that it was not always in relation to menstruation. It is easy to appreciate how the importance of cramping to patients with PCOS may have been underestimated by clinicians, given that women without PCOS also often experience painful cramping with menstruation. Both the greater intensity with which PCOS-related cramping is experienced, and the potential for it to occur in the absence of menstruation, distinguish cramping as a PCOS symptom that warrants consideration for medical management and the measurement of treatment benefit.

After cramping, the symptom concepts most frequently expressed by patients were about facial hair, irregular menstruation, and heavy bleeding. Each of these symptoms has been described in other qualitative interview studies in relation to the patient experience with PCOS [8–16, 31, 32], but only facial hair (hirsutism) and irregular menstruation are specifically included in the PCOS-Q and PCOSQ-50. Heavy bleeding was also not mentioned during the interviews with clinical experts as a symptom of importance. Thus, bleeding intensity, not just frequency of menstruation, may be another important aspect of patient experience with PCOS that is currently underappreciated.

Infertility was only the fifth most frequently reported symptom concept among patients with PCOS, but it was one of the most bothersome symptoms (second only to weight gain) when taking into account both the mean score (8.9) and the number of patients who rated it (*n* = 15). Other symptoms that were not predominant in terms of the frequency of their expression, but which were clearly important to patients in terms of the bother they caused, were weight gain and difficulty losing weight. These results highlight the limitations of examining the frequency of patient language alone, and the importance of combining patient language with other measures to achieve a multidimensional picture of the patient’s experience of a disease.

The most frequently expressed impact concepts were those relating to emotional well-being and functioning, which was consistent with clinicians’ views about the general impacts of PCOS and the well-established role of this impact in the literature [8–10, 12, 14, 15, 18, 31]. The second most predominant impact was compensatory behaviors, which was also highlighted by clinicians as a key impact of PCOS and has also been described as a key theme in qualitative studies [9, 18]. The coping behaviours described by patients with PCOS were usually in relation to managing physical appearance, such as weight (dietary changes, increased exercise), facial and body hair (medication use, shaving, trimming, plucking), and skin problems (medication use). Coping behaviors can hide the true impact of some symptoms. For example, the use of medication was a frequently reported impact and was often used to reduce the appearance of symptoms of PCOS such as acne, darkened skin, and unwanted hair. The frequent use of medication to hide these symptoms may thus be a proxy indicator of their importance to patients. Similarly, the absence of items covering coping behaviours both the PCOSQ and PCOSQ-50 may mean that the impact of some symptoms is underestimated.

Obstructive sleep apnea is far more prevalent among women with PCOS versus the general population than would be expected by chance alone, and weight-related issues in patients with PCOS probably contribute to this association [33–35]. It is therefore not surprising that sleep apnea was viewed by clinicians as an important impact of PCOS. However, sleep apnea was not frequently expressed as an impact of PCOS by the patients themselves. Although reasons for sleep- and energy-related problems other than sleep apnea were reported by patients and are obviously important, it is also possible that sleep apnea was underreported by patients with PCOS because many of them are unaware that they have it. Indeed, a recent study showed that more than 90% of physicians who manage patients with PCOS rarely ordered a sleep study, which is required for the diagnosis of sleep apnea [36].

It should be reiterated that the population used in our study excluded patients who had menstruated in the previous month. It is possible that this exclusion criterion selected for patients with a different symptom profile to the ‘typical’ PCOS population. However, any such effect is likely to be small, since irregular periods are part of the Rotterdam criteria used in the clinic to diagnose PCOS. Therefore, this exclusion criterion would merely select for individuals who happened to be experiencing a lack of menstruation in the month before study recruitment, rather than a sub-population of patients with distinct PCOS symptomatology. It is worth noting that, compared with purely thematic analyses that have constituted the bulk of qualitative studies performed in patients with PCOS, the frequency of concept expression is a more objective measure of the relative importance of concepts expressed by patients. This may be why novel insights into the patient experience with PCOS were gained in our study, although other differences in study design cannot be ruled out, such as the recruitment setting (primary care treatment clinics) and bespoke interview guide.

## Conclusions

The results of our study indicate that currently available PCOS-specific PROs are not optimal for use in trials of drugs aimed at targeting the underlying cause of PCOS, as they do not include a number of key symptoms and impacts of importance to these patients. In addition, some PCOS symptoms may be underappreciated in the clinic.

## Additional files

Additional file 1: Interview guide. (PDF 498 kb)

Additional file 2: Summary of the literature used to develop the draft PCOS disease model. (DOCX 29 kb)

Additional file 3: Draft PCOS disease model. (DOCX 182 kb)

## Abbreviations

PCOS: Polycystic ovary syndrome
PCOSQ: Polycystic ovary syndrome questionnaire
PRO: Patient reported outcome
SD: Standard deviation
