# Supplementary material for: Understanding polycystic ovary syndrome from the patient perspective: a concept elicitation patient interview study
Source: Health Qual Life Outcomes. 2017 Aug 18;15:162. doi: 10.1186/s12955-017-0736-3 (PMC5562990; doi:10.1186/s12955-017-0736-3)
Supplement: Supplementary file 1 — Interview guide. (PDF 498 kb) [file 12955_2017_736_MOESM1_ESM.pdf]

Day	Month	Year	Site Code	Subject ID Number

INTERVIEW GUIDE
for Concept Elicitation Interviews

Study Objectives	Progression of Questions in Interview Guide
**Overall Goal of the Study:** To understand the signs/symptoms and impacts that are relevant to patients with polycystic ovary syndrome (PCOS)<br><br>**Primary interview objectives:**<br><br>a) To understand the day-to-day experience of PCOS symptoms<br><br>b) To understand the day-to-day symptom related impacts of PCOS<br><br>c) To understand, in depth, the experience of the PCOS symptoms and impacts.<br><br>d) To understand the experiences and expectations related to PCOS treatment	**OVERALL FLOW:**<br><br>**Proximal:** Symptoms and signs explored thoroughly<br>⇩<br>**Distal:** Impacts (as they relate to symptoms or signs clusters)<br>⇩<br>Ratings, Descriptive data<br><br>***Within all Sections:*** Spontaneous first, probe specific territories for completion after that, and follow with worksheets or exercises at the close of each section (symptoms, signs, impacts, etc.)<br><br>**MAIN QUESTIONS IN INTERVIEW GUIDE:**<br><br>1. Which PCOS symptoms do patients have? (How do they describe their PCOS symptoms?)<br>2. How have the symptoms changed over time? What are a typical and an atypical month like?<br>3. What are the specific characteristics of the symptoms described by the patient?<br>4. What impacts do patients experience because of the symptoms? How has their condition limited their ability to do daily activities?<br>5. How do patients experience the PCOS treatment?

Day	Month	Year	Site Code	Subject ID Number

INTERVIEW GUIDE
for Concept Elicitation Interviews

TO BEGIN:

1. Introduce yourself and your association with the study
2. Explain purpose of the interview
3. Thank her for willingness to participate
4. Assure her of confidentiality
5. Let her know the general process of how it will proceed

The Primary Objective of this Interview:

This interview will take approximately 60 minutes. I will be asking you a variety of questions that will help me to better understand your experience with polycystic ovary syndrome, and any impacts it may have had on your daily life. I will be asking about:

- The symptoms you experience as a result of the polycystic ovary syndrome.
- The ways this limits you in day-to-day life.
- Your experiences and expectations with regards to treatment

How this Interview will work:

I will be asking general questions about the polycystic ovary syndrome, and after each one there will be some time for you to respond. Please respond with whatever is on your mind.

To begin the interview, I want to remind you that it is being recorded. I will try to not use your name from the point that I turn the recorder on, and I will ask you to try and not use your name or the names of friends or family in any of your responses. This will help keep the interview anonymous.

I will only be using the recording to remind me of the important things you said so we can represent everything more accurately at the end of the study. The recordings will be transcribed, and then put together with transcripts from everyone else taking part in these interviews. You won't be able to be individually identified in any of the reports that result from these interviews. This study information will be used to help make better decisions about how to measure these important issues in future studies.

Do you have any questions before we start?

Day	Month	Year	Site Code	Subject ID Number

CONCEPT ELICITATION INTERVIEW ITEMS

[Interviewer]: Be prepared to note symptoms and impacts offered by patient in their exact words in sections of this interview guide. Also be sure to document the indications of spontaneously offered versus probed symptoms and impacts and complete the various check boxes and lines that need completing as you go through this interview guide.

Before we start, I want to just check with you about how familiar you are with some words that we will be using throughout the interview. I'm going to be using the term "Symptom" a lot in the first few questions so I'd like to know what the term "symptom" means to you. How would you describe in your own words what a "symptom" is?

[Interviewer]: Wait for patient response, if patient does not understand symptom, use the following:

When I use the term "symptom" in the interview questions, I will be referring mostly to physical sensations or physical problems, although it's possible to have emotional symptoms as well. [Interviewer]: Be sure they have no questions before you continue.

Now, I would like to know what the term "polycystic ovary syndrome" means to you.

- Are you familiar with this term?
- What do your family and friends know about your condition? How do you talk to them about your PCOS?
- Have any of your relatives ever been diagnosed with PCOS? If so, how do they relate to you?

Day	Month	Year	Site Code	Subject ID Number

FIRST DISCUSSION ITEM:

*(**Purpose:** To settle the subject and locate them in the desired territory)*

To start, can you tell me a little bit about the time when you started experiencing symptoms related to the polycystic ovary syndrome?

- How long ago were you diagnosed with PCOS? [Enter # years/months: ____/____]
- How did you find out you had PCOS? Who diagnosed you? Where you referred to a specialist at that time?
- What were you feeling at that time?
- How would you describe the symptoms you felt at that time?
- Please describe how you feel nowadays
- Can you describe any physical sensations you had that were related to your PCOS?

SECOND DISCUSSION ITEM:

*(**Purpose:** To focus patient on the PCOS symptoms they experience and get a description of how those have changed over time and how long have they experience these symptoms)*

[Interviewer]: Note symptoms she has mentioned so far
[Interviewer]: As the patient offers each new symptom, write it in her exact words in the symptom probe segments on forward pages where the individual symptom probes appear.

So, you have just mentioned that you felt ____, ____, ____ when the PCOS first started. Can you talk a little more about these symptoms?

- Can you describe how often these symptoms appear?
- Is there something regular about when they appear or what causes them?
- What makes them better? What makes them worse?
- Can you describe how these symptoms have changed over time? *(From when they first started to happen until now?)*
- Did any of your symptoms go away over time? Were there any new symptoms that appeared over time?
- Looking back over the course of events since these difficulties started, how would you describe your worst symptom?
- Is that symptom still your worst symptom now? (*This week*)

Day	Month	Year	Site Code	Subject ID Number

THIRD DISCUSSION ITEM:

*(**Purpose:** To focus patient on specific symptoms experienced, to get a description of a typical day and the variation of symptoms over time)*

I would like to focus on how your PCOS symptoms are now. To do that, I need to ask you to pick a recent day when you definitely experienced symptoms related to your condition. This should be a day you feel you can remember fairly well.

[Interviewer]: The day selected must be as recent as possible.
Can you pick a recent day to talk about and tell me what that day was?
[Interviewer]: Write day: __________

Ok, thinking about that day, ___________ [say the day it was]

- Can you describe the very first symptom you remember having right after you woke up that morning?
- What other symptoms did you have as you went through your morning routine on ________[say day]
- What other symptoms did you notice that day before lunch time?
- Did you notice any other symptoms as you went through the afternoon that day? [Describe]
- Can you describe any symptoms you had around dinner time on _______[say day]
- What about symptoms before going to bed?
- Do you remember experiencing any symptoms during the night?
- Can you describe how your sleep was that night?

We have just been talking about ______[say day]. **How typical was this day for you in terms of the symptoms you experience related to PCOS?**

- Would you call the day you just described better than usual? Fairly usual? Or worse than usual?
- In a usual week how many days like this do you have?
- How many days would be better than this one?
- Thinking just about the pain symptoms, what makes a good day?
- How would that change for a bad day? What would the main difference be?
- What helps you ease your symptoms on very bad days?
- Have you had periods of time where you were completely symptom free? How long were they?
- Do your symptoms tend to happen around a particular time of day?

Day	Month	Year	Site Code	Subject ID Number

FOURTH DISCUSSION ITEM:

*(**Purpose:** To get description of a typical month and the variation of symptoms over time)*

I'd like to ask you to think about the past six months and your PCOS. Was there one month that stands out as a fairly typical month where you definitely experienced symptoms related to PCOS. This should be a month you feel you can remember fairly well.

Once you pick the month you want to talk about, let me know which one it is.

[Interviewer]: Write month: ___________

Ok, thinking about that month, ____________ [say the month it was]

- Can you describe the first symptom you remember having in _______(say month)
- What other symptoms did you have during the first weeks of _______(say month)
- Did any new symptoms appear during the last weeks of ___________(say month)

We have just been talking about _____[say month]. **How typical was this month for you in terms of the symptoms you experience related to PCOS?**

- Would you call the month you just described better than usual? Fairly usual? Or worse than usual?
- In any given year how many months like this one do you have?
- How many months would be better than this one?
- How would that change for a bad month? What would the main difference be?
- What helps you ease your symptoms on very bad months?
- Have you had periods of time where you were completely symptom free? How long were they?
- Do your symptoms tend to happen around a particular time of the month?

Day Month Year Site Code Subject ID Number

FIFTH DISCUSSION ITEM:

*(**Purpose:** To make sure all the PCOS symptoms the patient experiences have come out for discussion)*

As you have been talking, I have been writing down the different symptoms you have talked about. The ones you have mentioned so far are: ____, ____, ____ [read symptoms so far].

Are there <u>any other symptoms</u> that you have that might be related to the polycystic ovary syndrome?

[Interviewer]:

- Complete the table for the ones they have already mentioned first. **Mark S/P box for each**
- **Read any to them that they have <u>not already mentioned</u>.**
- Circle which symptom in each group that you inquire about
- Add any new symptoms patient has to offer spontaneously (Mark S/P box for each line)
- **Update the symptom probe sections on following pages once symptom picture is complete**

Day		Month			Year					Site Code				Subject ID Number			

Now I'm going to describe some additional symptoms that some women with PCOS describe. As I read this list, please tell me if you recognize experiencing any of these

	Spontaneously offered (✓)	Recognized Probe (✓)	Not Affected (✓)	Symptom Description
**A.**				**Irregular menstrual periods**
**B.**				**No Menstrual periods**
**C.**				**Menstrual Pain**
**D.**				**Bleeding**<br>*Heavy bleeding/flow*<br>*Long duration*
**E.**				**Bloating**
**F.**				**Infertility**<br>*Inability to conceive a baby*<br>*Inability to carry a pregnancy to full term*
**G.**				**Anovulation**<br>*Ovulation does not occur*
**H.**				**Hirsutism**<br>*Unwanted additional facial hair*<br>*Unwanted additional body hair*
**I.**				**Alopecia**<br>Hair loss in your head
**J.**				**Obesity**
**K.**				**Rapid increase in weight**
**L.**				**Difficulty losing weight**
**M.**				**Fluctuating weight**<br>*Weight that goes up and down*
**N.**				**Bodily pain**<br>Part of the body ___________________
**O.**				**Acne**
**P.**				**Other**

Day	Month	Year	Site Code	Subject ID Number

SIXTH DISCUSSION ITEM:

*(**Purpose:** To make a consistent exploration around the patient experiences related to having irregular menstrual periods)*

[Interviewer]: Ask these questions if the patient acknowledges having irregular menstrual periods.

Ok, you mentioned before that you have irregular menstrual periods. I would like you to describe in more detail what this concept means to you.

- What do you consider to be a normal menstrual period? How does this differ from your own cycle?
- What is your main concern when it comes to having irregular periods?

SEVENTH DISCUSSION ITEM:

*(**Purpose:** To make a consistent exploration around the pain that the patient experiences)*

[Interviewer]: Ask these questions if the patient acknowledges having pain.

Ok, you mentioned before that you experience pain related to your PCOS.

- Where do you feel the pain? In which part(s) of your body?
- Can you describe the pain?
- Does it change throughout the day or month? Does it come and go?

EIGHTH DISCUSSION ITEM:

*(**Purpose:** To make a consistent exploration around the characteristics of the most relevant symptoms that the patient experiences)*

[Interviewer]: Use patient language for each symptom written into probe segments below. If during the last item, patient responds that one symptom you asked about is the same as another one they have already given, put that description next to their symptom language below in parentheses. *(We are not probing on similarity of language but will capture it if it comes up.)*

Next I'm going to ask you some very specific questions about each of the symptoms you have been talking about and that we did not discussed in the previous section.

Day	Month	Year	Site Code	Subject ID Number

A: [write in symptom]_____________________☐**spontaneously** offered ☐**probed** response

(Use patient's language)

Mark each ☑ when asked Strike through the box ☐ if there was a reason to not ask the probe for this symptom

☐ What triggers the _________ (**symptom**)?

☐ When you have this symptom, how bad (severe) is it when it's at its worst? ☐

(On a scale of 0 =none to 10=extremely severe)

☐ Does it change throughout the day/month? (*Does it come and go? How often?)*

☐ How often do you usually have this symptom? *(Daily? Weekly? Monthly? Less than that?)*

☐ How long does it usually last when it happens?

☐ How do you know when it gets better? What changes?

☐ Is there anything you can do to feel better?

B: [write in symptom]_____________________☐**spontaneously** offered ☐**probed** response

(Use patient's language)

Mark each ☑ when asked Strike through the box ☐ if there was a reason to not ask the probe for this symptom

☐ What triggers the _________ (**symptom**)?

☐ When you have this symptom, how bad (severe) is it when it's at its worst? ☐

(On a scale of 0 =none to 10=extremely severe)

☐ Does it change throughout the day/month? (*Does it come and go? How often?)*

☐ How often do you usually have this symptom? *(Daily? Weekly? Monthly? Less than that?)*

☐ How long does it usually last when it happens?

☐ How do you know when it gets better? What changes?

☐ Is there anything you can do to feel better?

Day	Month	Year	Site Code	Subject ID Number

C: [write in symptom]___________________☐**spontaneously** offered ☐**probed** response

(Use patient's language)

Mark each ☑ when asked Strike through the box ☐ if there was a reason to not ask the probe for this symptom

☐ What triggers the _________ (**symptom**)?

☐ When you have this symptom, how bad (severe) is it when it's at its worst? ☐

(On a scale of 0 =none to 10=extremely severe)

☐ Does it change throughout the day/month? (*Does it come and go? How often?)*

☐ How often do you usually have this symptom? *(Daily? Weekly? Monthly? Less than that?)*

☐ How long does it usually last when it happens?

☐ How do you know when it gets better? What changes?

☐ Is there anything you can do to feel better?

D: [write in symptom]___________________☐**spontaneously** offered ☐**probed** response

(Use patient's language)

Mark each ☑ when asked Strike through the box ☐ if there was a reason to not ask the probe for this symptom

☐ What triggers the _________ (**symptom**)?

☐ When you have this symptom, how bad (severe) is it when it's at its worst? ☐

(On a scale of 0 =none to 10=extremely severe)

☐ Does it change throughout the day/month? (*Does it come and go? How often?)*

☐ How often do you usually have this symptom? *(Daily? Weekly? Monthly? Less than that?)*

☐ How long does it usually last when it happens?

☐ How do you know when it gets better? What changes?

☐ Is there anything you can do to feel better?

Day	Month	Year	Site Code	Subject ID Number

E: [write in symptom]______________________ ☐**spontaneously** offered ☐**probed** response
(Use patient's language)

Mark each ☑ when asked   Strike through the box ☐ if there was a reason to not ask the probe for this symptom

☐ What triggers the __________ (**symptom**)?
☐ When you have this symptom, how bad (severe) is it when it's at its worst? ☐
(On a scale of 0 =none to 10=extremely severe)
☐ Does it change throughout the day/month? (*Does it come and go? How often?)*
☐ How often do you usually have this symptom? *(Daily? Weekly? Monthly? Less than that?)*
☐ How long does it usually last when it happens?
☐ How do you know when it gets better? What changes?
☐ Is there anything you can do to feel better?

F: [write in symptom]______________________ ☐**spontaneously** offered ☐**probed** response
(Use patient's language)

Mark each ☑ when asked   Strike through the box ☐ if there was a reason to not ask the probe for this symptom

☐ What triggers the __________ (**symptom**)?
☐ When you have this symptom, how bad (severe) is it when it's at its worst? ☐
(On a scale of 0 =none to 10=extremely severe)
☐ Does it change throughout the day/month? (*Does it come and go? How often?)*
☐ How often do you usually have this symptom? *(Daily? Weekly? Monthly? Less than that?)*
☐ How long does it usually last when it happens?
☐ How do you know when it gets better? What changes?
☐ Is there anything you can do to feel better?

Day	Month	Year	Site Code	Subject ID Number

SYMPTOM WORKSHEET:

*(**Purpose:** To obtain a bothersome rating for each symptom)*

The worksheet I am giving you now has a list of the different symptoms you have described as we have been talking.

Can you please have a look at this list to be sure that you agree with how I have them written?

- Did I get them all? Are there others you want to add?
- Is the wording correct for the ones I have listed?
- Do you want to change anything about the list?

[Interviewer]: Once patient agrees to list:

For each symptom listed, go across to the numbered scale and circle the number that you feel best represents how much that particular symptom bothers you.

A zero would mean you are not bothered by it at all. A 10 would mean it bothers you an extreme amount.

[Interviewer]: Be sure patient clearly understands task and let them continue to do the worksheet on their own. **Use the time** to write the symptoms on the cards for the importance ranking exercise.

SYMPTOM WORKSHEET [USE ACTUAL FULL SHEET HANDOUT]

Please review each symptom listed below and rate how much it bothers you.

Column 1	Column 2
**SYMPTOMS**	***Please circle one number below***<br>*(0=not bothersome at all)*<br>*(10=extremely bothersome)*
	0 1 2 3 4 5 6 7 8 9 10
	0 1 2 3 4 5 6 7 8 9 10
	0 1 2 3 4 5 6 7 8 9 10

Day Month Year Site Code Subject ID Number

NINTH DISCUSSION ITEM:

*(**Purpose:** To fully identify PCOS symptom related impacts)*

So far we have been talking about the symptoms related to your PCOS. This next section changes the focus over to how your everyday life is affected by PCOS.

- Where are the places in your life where the polycystic ovary syndrome affects you?
- What activities have you had to cut back on because of the PCOS?
- What types of activities would you do if you didn't have PCOS?
- How would you describe your relationship with others since your difficulties with PCOS?

[Interviewer]:
(1) Give patient time first to give spontaneous responses, indicate those.
(2) Then probe remaining areas, indicate those and mark the ones patient does not experience.
(3) Then add any impacts the patient brings up that are not on the list and mark them 'Spontaneous'.

THEN: Read any of the impacts mentioned below that have <u>NOT ALREADY been mentioned</u> by the patient]

Now I'm going to describe some other ways in which PCOS can affect people's life. As I read this list, please tell me if you recognize any of these.

Day Month Year Site Code Subject ID Number

	Spontaneously offered (✓)	Recognized Probe (✓)	Not Affected (✓)	Area of Impact
**A.**				**Sleep apnea**<br>Sleep problems?<br>( ) *One or more pauses in breathing or shallow breaths while you sleep*
**B.**				**Changes in Energy/ Vitality**<br>( ) *Decreased*<br>( ) *Lack of energy*
**C.**				**Impaired sexual functioning**<br>*Sexual problems*
**D.**				**Daily activities**<br>( ) *Working*<br>( ) *Studying*<br>( ) *Eating*
**E.**				**Emotional functioning**<br>( ) *Altered self-perception* ( ) *Anxiety*<br>( ) *Emotional fluctuations* ( ) *Embarrassment*<br>( ) *Negative self-image* ( ) *Depression*
**F.**				**Worry about related diseases**<br>( ) *Hyperinsulinaemia* - Excess of insulin relative to glucose<br>( ) *Periodontal disease* – Inflammation of the tissues around the teeth<br>( ) *Type 2 diabetes* – diabetes in the future<br>( ) *Cardiovascular disease* – heart disease in the future<br>( ) *Recurrent pregnancy loss* - Pregnancy<br>( ) Increased risk for gestational diabetes - Pregnancy
**G.**				**Social functioning**<br>( ) *Relationship with spouse affected*<br>( ) *Family relations affected*<br>( ) *Relations with friends and acquaintances affected*<br>( ) *Workplace relations affected*
**H.**				**Compensatory behaviors:**<br>( ) *Waxing facial/body hair,*<br>( ) *Acne medications*<br>( ) *Exercise because of weight fluctuations*<br>( ) *Diet because of weight fluctuations.*
**I.**				**Other?**<br>Describe _________________

Day Month Year Site Code Subject ID Number

TENTH DISCUSSION ITEM

***(Purpose:** To make a consistent exploration around the characteristics of the most relevant symptom related impacts patient experiences)*

[Interviewer]: Use patient language for each impact written into probe segments below. If during the last item, patient responds that one impact you asked about is the same as another one they have already given, put that description next to their symptom language below in parentheses. *(We are not probing on similarity of language but will capture it if it comes up.)*

Next I'm going to ask you some very specific questions about each of the impacts you have been talking about.

A: [write in impact]______________________ ☐**spontaneously** offered ☐**probed** response
(Use patient's language)

Mark each ☑ when asked Strike through the box ⊟ if there was a reason to not ask the probe for this symptom

☐ How often do you usually experience this impact? *(Monthly? Daily? Less than that?)*
☐ How long have you been experiencing this impact?
☐ Is there anything that makes this impact more difficult to handle? What changes?
☐ Is there anything that makes this impact less difficult to handle? What changes?

B: [write in impact]______________________ ☐**spontaneously** offered ☐**probed** response
(Use patient's language)

Mark each ☑ when asked Strike through the box ⊟ if there was a reason to not ask the probe for this symptom

☐ How often do you usually experience this impact? *(Monthly? Daily? Less than that?)*
☐ How long have you been experiencing this impact?
☐ Is there anything that makes this impact more difficult to handle? What changes?
☐ Is there anything that makes this impact less difficult to handle? What changes?

Day	Month	Year	Site Code	Subject ID Number

C: [write in impact]_______________________ ☐**spontaneously** offered ☐**probed** response

(Use patient's language)

Mark each ☑ when asked *Strike through the box ☐ if there was a reason to not ask the probe for this symptom*

☐ How often do you usually experience this impact? *(Monthly? Daily? Less than that?)*

☐ How long have you been experiencing this impact?

☐ Is there anything that makes this impact more difficult to handle? What changes?

☐ Is there anything that makes this impact less difficult to handle? What changes?

D: [write in impact]_______________________ ☐**spontaneously** offered ☐**probed** response

(Use patient's language)

Mark each ☑ when asked *Strike through the box ☐ if there was a reason to not ask the probe for this symptom*

☐ How often do you usually experience this impact? *(Monthly? Daily? Less than that?)*

☐ How long have you been experiencing this impact?

☐ Is there anything that makes this impact more difficult to handle? What changes?

☐ Is there anything that makes this impact less difficult to handle? What changes?

E: [write in impact]_______________________ ☐**spontaneously** offered ☐**probed** response

(Use patient's language)

Mark each ☑ when asked *Strike through the box ☐ if there was a reason to not ask the probe for this symptom*

☐ How often do you usually experience this impact? *(Monthly? Daily? Less than that?)*

☐ How long have you been experiencing this impact?

☐ Is there anything that makes this impact more difficult to handle? What changes?

☐ Is there anything that makes this impact less difficult to handle? What changes?

Day Month Year Site Code Subject ID Number

F: [write in impact]_______________________☐**spontaneously** offered ☐**probed** response
(Use patient's language)

Mark each ☑ when asked Strike through the box ⊟ if there was a reason to not ask the probe for this symptom

☐ How often do you usually experience this impact? *(Monthly? Daily? Less than that?)*
☐ How long have you been experiencing this impact?
☐ Is there anything that makes this impact more difficult to handle? What changes?
☐ Is there anything that makes this impact less difficult to handle? What changes?

G: [write in impact]_______________________☐**spontaneously** offered ☐**probed** response
(Use patient's language)

Mark each ☑ when asked Strike through the box ⊟ if there was a reason to not ask the probe for this symptom

☐ How often do you usually experience this impact? *(Monthly? Daily? Less than that?)*
☐ How long have you been experiencing this impact?
☐ Is there anything that makes this impact more difficult to handle? What changes?
☐ Is there anything that makes this impact less difficult to handle? What changes?

H: [write in impact]_______________________ ☐**spontaneously** offered ☐**probed** response
(Use patient's language)

Mark each ☑ when asked Strike through the box ⊟ if there was a reason to not ask the probe for this symptom

☐ How often do you usually experience this impact? *(Monthly? Daily? Less than that?)*
☐ How long have you been experiencing this impact?
☐ Is there anything that makes this impact more difficult to handle? What changes?
☐ Is there anything that makes this impact less difficult to handle? What changes?

Day	Month	Year	Site Code	Subject ID Number

ELEVENTH DISCUSSION ITEM

Purpose: To get a description of how the negative self-image impacts the patient overall functioning

Interviewer]: Ask these questions if the patient acknowledges having PCOS symptoms that impact her self-image.

Ok, you mentioned before that you experience _________(say patient words describing low self-image) related to your PCOS.

- How has this affected your daily life? (typical activities, work behavior,)
- How has this affected other areas of your life? (social life, family life, sex life)
- Has this changed throughout time?

IMPACT WORKSHEET:

(Purpose: To obtain a difficulty rating for each impact)

Now, as you have been talking, I have listed the different descriptions you have been giving about how having PCOS has affected your life.

Can you please have a look at this list to be sure that you agree with how I have them written?

- Did I get them all? Are there others you want to add?
- Is the working correct for the ones I have listed?
- Do you want to change anything about the list?

[Interviewer]: Once patient agrees to list:

For each one of these, go across to the numbered scale and circle the number that shows how difficult each impact is for you to cope with.

A zero would mean you don't find it difficult at all. A 10 would mean it's extremely difficult to cope with.

[Interviewer]: Be sure patient clearly understands task and let them continue to do the worksheet on their own.

Day	Month	Year	Site Code	Subject ID Number

IMPACT WORKSHEET [USE ACTUAL FULL SHEET]

Please review each impact listed below and rate how difficult it is to cope with.

Column 1	Column 2
**IMPACTS**	***Please circle one number below***<br>*(0=not difficult at all)*<br>*(10=extremely difficult)*
	0 1 2 3 4 5 6 7 8 9 10
	0 1 2 3 4 5 6 7 8 9 10
	0 1 2 3 4 5 6 7 8 9 10

TWELFTH DISCUSSION ITEM

Purpose*: To get a description of treatment related experiences and treatment goals*

We are almost done now. So far we have been talking about the symptom related impacts of your polycystic ovary syndrome. This last next section changes the focus to your treatment.

- What is your current treatment for PCOS?
- How long have you been on this current PCOS treatment?
- Do you believe your symptoms of PCOS have improved with this treatment?
- If yes, which symptoms have improved?
- For each symptom that has improved:
- How long after you started your treatment did you notice any improvement in [symptom]? In other words, how long after starting your current treatment did that symptom start to get better?
- How soon after starting treatment did that symptom go away or get as good as you would like it to be?

  How satisfied are you with your current treatment? Why?
- On a scale from 0 to 10, please rate how satisfied you are with your current treatment. A **zero** would mean you are not satisfied at all and a **10** would mean you are extremely satisfied
- Are you experiencing any negative effects or adverse events from your treatment?
- What negative effects do you consider tolerable?
- What other treatments for PCOS have you had? How satisfied were you with these treatments? Why?

Day Month Year Site Code Subject ID Number

I would now like to ask you about your treatment goals.

- How can you tell if your treatment is working?
- If you could choose which aspect of your symptoms to improve first; would it be how bad they are? How long they last? Or how often you have them? Why?
- Which PCOS symptom do you think is the most important for a treatment to improve?
- How would you define a successful treatment?
- How would it look like?
- How would you define a moderately successful treatment?
- How would it look like?
- How would a moderately successful treatment be different from an unsuccessful treatment?
- What do you expect from a treatment for menstrual problems? (Ask ONLY patients with Irregular Menstruation)
- Are you actively trying to get pregnant?

Thank you for your participation today!

Day	Month	Year	Site Code	Subject ID Number

SYMPTOM WORKSHEET

Please review each symptom listed below and rate how much it bothers you.

Column 1	Column 2
**SYMPTOMS**	***Please circle one number below*** *(0=not bothersome at all) (10=extremely bothersome)*
	0 1 2 3 4 5 6 7 8 9 10
	0 1 2 3 4 5 6 7 8 9 10
	0 1 2 3 4 5 6 7 8 9 10
	0 1 2 3 4 5 6 7 8 9 10
	0 1 2 3 4 5 6 7 8 9 10
	0 1 2 3 4 5 6 7 8 9 10
	0 1 2 3 4 5 6 7 8 9 10
	0 1 2 3 4 5 6 7 8 9 10
	0 1 2 3 4 5 6 7 8 9 10
	0 1 2 3 4 5 6 7 8 9 10
	0 1 2 3 4 5 6 7 8 9 10
	0 1 2 3 4 5 6 7 8 9 10
	0 1 2 3 4 5 6 7 8 9 10

Day	Month	Year	Site Code	Subject ID Number

IMPACT WORKSHEET

Please review each impact listed below and rate how difficult it is to cope with.

**Column 1**	**Column 2**
**IMPACTS**	***Please circle one number below*** *(0=not difficult at all) (10=extremely difficult)*
	0 1 2 3 4 5 6 7 8 9 10
	0 1 2 3 4 5 6 7 8 9 10
	0 1 2 3 4 5 6 7 8 9 10
	0 1 2 3 4 5 6 7 8 9 10
	0 1 2 3 4 5 6 7 8 9 10
	0 1 2 3 4 5 6 7 8 9 10
	0 1 2 3 4 5 6 7 8 9 10
	0 1 2 3 4 5 6 7 8 9 10
	0 1 2 3 4 5 6 7 8 9 10
	0 1 2 3 4 5 6 7 8 9 10
	0 1 2 3 4 5 6 7 8 9 10
	0 1 2 3 4 5 6 7 8 9 10
	0 1 2 3 4 5 6 7 8 9 10
	0 1 2 3 4 5 6 7 8 9 10
	0 1 2 3 4 5 6 7 8 9 10
	0 1 2 3 4 5 6 7 8 9 10
	0 1 2 3 4 5 6 7 8 9 10
