# Supplementary material for: Understanding polycystic ovary syndrome from the patient perspective: a concept elicitation patient interview study
Source: Health Qual Life Outcomes. 2017 Aug 18;15:162. doi: 10.1186/s12955-017-0736-3 (PMC5562990; doi:10.1186/s12955-017-0736-3)
Supplement: Supplementary file 2 — Summary of the literature used to develop the draft PCOS disease model. (DOCX 29 kb) [file 12955_2017_736_MOESM2_ESM.docx]

**Table -** *Summary of the literature used to develop the draft disease model*

| **Citation** | **Reference** | **Description/Summary results** |
| --- | --- | --- |
| Billhult & Stener-Victorin, (2012) | Billhult A, Stener-Victorin E. Acupuncture with manual and low frequency  electrical stimulation as experienced by women with polycystic ovary syndrome: a qualitative study. BMC Complement Altern Med. 2012 3; 12:32. | In this study, eight women with PCOS were interviewed following repeated acupuncture treatments. The objective of the study was to describe the experience of acupuncture treatment.  One of the participants who expected results from the treatment to affect, for example, her menstruation cycle expressed the following:  “My hair growth decreased, and that was... I had never hoped for that...”  An effect of acupuncture treatment such as regaining a regular menstrual period provided relief for the participants. Even though menstruation was experienced as bothersome, it felt good to know the body was working in that sense. “It is just a relief to know that it [the menstruation] arrives every month. Even if that week is not a fun one...it is good to know that it will be there”  Several of the participants expressed a feeling of wellness strongly connected to bodily functions such as being able to get pregnant, decreased hair growth and smooth skin.  Besides effects on menstruation, the women in the present study also experienced effects such as decreased hair growth, decreased cravings for sweets and decreased acne according to the interviews. At follow-up, 4 months after the last treatment, women receiving acupuncture rated less acne. |
| Cela et al. (2003) | Cela E, Robertson C, Rush K, Kousta E, White DM, Wilson H, Lyons G, Kingsley  P, McCarthy MI, Franks S. Prevalence of polycystic ovaries in women with  androgenic alopecia. Eur J Endocrinol. 2003;149(5):439-42 | Cela et al., 2003 examined the strength of the association between androgenic alopecia and PCOS. They examined the prevalence of ultrasound-based polycystic ovarian morphology and associated clinical and biochemical features in a large multiethnic group of women whose presenting complaint was of alopecia, and in a control group.  Among the 89 women studied, women with alopecia had a higher prevalence of PCOS and hirsutism than the control population. Findings confirmed an association between androgenic alopecia and PCOS, and other symptoms of hyperandrogenaemia. Thus, most women who present with androgenic alopecia as their primary complaint also have PCOS and have indices of abnormal androgen production. |
| Cinar et al. (2012) | Cinar N, Harmanci A, Demir B, Yildiz BO. Effect of an oral contraceptive on  emotional distress, anxiety and depression of women with polycystic ovary  syndrome: a prospective study. Hum Reprod. 2012; 27(6):1840-5. | In a prospective observational study, Cinar et al., 2012 aimed to determine the impact of an oral contraceptive treatment (ethinyl estradiol drospirenone) on health-related quality of life, depression and anxiety symptoms in PCOS.  Participants completed PCOSQ, Beck Depression Inventory, Hospital Anxiety and Depression Scale and the General Health Questionnaire. Serum androgens, fasting insulin, fasting and postload glucose values during an oral glucose tolerance test were measured. Changes in these variables and the scores of questionnaires were evaluated after 6 months of treatment.  Menstrual and hirsutism problems were the most serious concerns, followed by emotional problems on the PCOSQ. 22.2% had clinical depression scores. After treatment, regular menstrual cycles were attained and hirsutism significantly improved in all patients. The hirsutism and emotion domains of the PCOSQ improved at 6 months. Overall, depression, anxiety mean scores and depression rates did not show a significant change. |
| Ciotta et al. (2001) | Ciotta L, Calogero AE, Farina M, De Leo V, La Marca A, Cianci A. Clinical,  endocrine and metabolic effects of acarbose, an alpha-glucosidase inhibitor, in PCOS patients with increased insulin response and normal glucose tolerance. Hum Reprod. 200; 16(10):2066-72. | Ciotta et al., 2001 evaluated whether treatment with acarbose improved hyperandrogenic symptoms (hirsutism and acne), insulin and androgen serum concentrations in hyperinsulinaemic patients with PCOS.  Hirsutism, and acne/seborrhea scores, hormonal and sex hormone binding globulin serum concentrations, glycaemia and insulin responses to glucose were measured before and after three months of treatment.  Ciotta et al., 2001 found that there was a significant reduction in acne/seborrhea score with patients treated with acarbose and 8 of the 30 women resumed a regular menstrual rhythm. The improvement in acne/seborrhea score was associated with a significant decrease of insulin response to oral glucose load and of LH and androgen serum concentrations and with a significant rise of sex hormones binding globulin concentration. |
| Crete & Adamshick (2011) | Crete J, Adamshick P. Managing polycystic ovary syndrome: what our patients are telling us. J Holist Nurs. 2011; 29(4):256-66. | The purpose of this study was to describe the lived experience of women with PCOS in the management of their disorder and the meaning of that experience for them. Individual, semi-structured interviews were completed with 10 participants who were diagnosed with PCOS and managed by a health care practitioner(s) within the past 5 years.  One area of frustration centered on interactions with health care providers, which was reflected in a lack of confidence in providers along with a sense of inattentiveness from them.  The same participant remarked on the health care provider’s inattentiveness to her troublesome symptoms of weight gain and hirsutism: I think he knows about it but he doesn’t take the time to ask . . . he never looked at them [whiskers]. He never asked, “Do you shave them?” He doesn’t say, “How’s your diet going or are you losing any weight?” I don’t think he’s interested.  One PCOS client, with the complaint of irregular menses, described the one-dimensional treatment she received: “I suffered from really bad headaches and so I didn’t want to go back on the pill, but that always seemed to be the quick fix just to regulate my period. So it was kind of setting the PCOS aside, we’ll just put you on the pill and then you you’ll be regular.”  Her description captures the emotional upheaval of unrelenting symptoms and the ordeal of what the problem is. “I still have a body image issue. You know how they call it the ‘bearded lady syndrome.’ Ladies years ago used to get it and they didn’t know what it was. They used to get whiskers and humps on their back and big round stomachs. If I remember what causes it I don’t get as upset. Sometimes I don’t feel like explaining to everybody around me why I have this look . . . I don’t feel like going through the ordeal of what the problem is*.*”  Participants’ lived experience included frustration over lack of symptom improvement, primarily with weight control and hirsutism. One participant also discussed persistent pain she endured for 11 years.  “I am still taking the medication and I’m still having the pains... I see no improvement except for the acne. The pain is still there, so am I supposed to live with this pain for the rest of my life? Even when I have sexual intercourse, I can’t even pull through because of the pain.”  One participant expressed distress at not knowing the full implications of PCOS on her overall health. “Nobody really managed it [PCOS] at all. They never told me . . . when I was diagnosed with PCOS, that yes, this is stopping you from conceiving.”  “The pain on my ovary made me see the doctor. I would of course go on the Internet and I’d find I think I have cancer. My mom said we were going to the doctor and everything was fine in the end, but 18 years old and you see what the pain means on the Internet.”  In one participant’s experience, the formal searching and reading was helpful to pull together the symptom picture that she had noticed in herself and verify its relationship to the PCOS diagnosis. “I’ve done all my own research and reading, and in retrospect I do have some of the other symptoms, but I did not know that that’s what they were until I read up on it. I do have some dark facial hair. I do have the weight gain problem. The hormonal up and down mood swings type thing. I have some of them and I didn’t realize they were connected until I was diagnosed.” |
| Cronin et al. (1998) | Cronin L, Guyatt G, Griffith L, Wong E, Azziz R, Futterweit W, Cook D, Dunaif  A. Development of a health-related quality-of-life questionnaire (PCOSQ) for  women with polycystic ovary syndrome (PCOS). J Clin Endocrinol Metab. 1998; 83(6):1976-87. | In the development of the PCOSQ, Cronin et al., 1998 used PCOS patients’ assessments of symptoms in an initial pool of 182 items potentially relevant to women with PCOS to establish a final set of items. Initial items were based on semi-structured interviews with PCOS patients, a survey of health professionals who worked closely with PCOS women, and a literature review.  During the item reduction phase of the development of the PCOSQ, Cronin et al., 1998 had one hundred women with PCOS complete a questionnaire which measured how frequently women labeled the item as a problem for them and the importance they attached to it.  The top ten symptoms with the most reported patient impact included;   - overweight, - growth of visible hair on face, - difficulties staying at a weight you would like, - trouble dealing with weight, - growth of visible hair on chin, - growth of visible hair on the upper lip, - growth of visible hair on body, - tire easily, - irregular menstrual periods, - and headaches. |
| Fogel et al. (2001) | Fogel RB, Malhotra A, Pillar G, Pittman SD, Dunaif A, White DP. Increased prevalence of obstructive sleep apnea syndrome in obese women with polycystic ovary syndrome. J Clin Endocrinol Metab. 2001; 86(3):1175-80. | To determine whether obese women with PCOS have an increased prevalence of sleep apnea compared with age and weight-matched reproductively normal women, Fogel et al., 2001 performed overnight polysomnography for determination of the apnea-hypopnea index (AHI) in 18 obese women with PCOS and age and weight-matched control women.  Women with PCOS had a higher AHI than controls. Women with PCOS were also more likely to suffer from symptomatic obstructive sleep apnea syndrome. AHI correlated with waist-hip ratio, serum testosterone, and unbound testosterone in women with PCOS. Obese women with PCOS are at increased risk of OSA when compared with matched reproductively normal women. |
| Harlow et al. (1998) | Harlow BL, Signorello LB, Hall JE, Dailey C, Komaroff AL. Reproductive correlates of chronic fatigue syndrome. Am J Med. 1998 28; 105(3A):94S-99S. | A case-control study was conducted to determine whether menstrual and gynecologic abnormalities precede the onset of chronic fatigue syndrome (CFS) in women with this disorder to a greater extent than that observed among healthy controls. Harlow et al., 1998 identified 150 women with CFS and a comparison group of 149 women. Women with CFS reported increased gynecologic complications and a lower incidence of premenstrual symptomatology.  Factors suggestive of abnormal ovarian function--such as a history of polycystic ovarian syndrome, hirsutism, and ovarian cysts--were reported more often in CFS cases compared with controls. Frequent anovulatory cycles due to ovarian  hyperandrogenism (PCOS) or hyperprolactinemia may increase risk for CFS through loss of the potential immunomodulatory effects of progesterone in the presence of continued estrogen production. Harlow et al., 1998 hypothesized that frequent anovulatory cycles due to PCOS and/or hyperprolactinemia may explain the increased reporting of gynecologic complications and the lower reported premenstrual symptomatology observed in women with CFS. |
| Jedel et al. (2010) | Jedel E, Waern M, Gustafson D, Landén M, Eriksson E, Holm G, Nilsson L, Lind AK, Janson PO, Stener-Victorin E. Anxiety and depression symptoms in women with polycystic ovary syndrome compared with controls matched for body mass index. Hum Reprod. 2010; 25(2):450-6. | Jedel et al., 2010 sought to compare symptoms of anxiety and depression in women with PCOS and controls matched for age, body weight and body mass index (BMI). Participants completed the self-reported versions of the Brief Scale for Anxiety (BSA-S) and Montgomery Asberg Depression Rating Scale (MADRS-S).  Women with PCOS had a higher BSA-S score compared with controls. They scored higher on four individual symptoms: reduced sleep, worry, phobias, and pain. In general, they found that several anxiety symptoms distinguished women with PCOS from a control group matched on BMI. |
| Jones et al. (2011) | Jones GL, Hall JM, Lashen HL, Balen AH, Ledger WL. Health-related quality of life among adolescents with polycystic ovary syndrome. J Obstet Gynecol Neonatal  Nurs. 2011 40(5):577-88. | In this qualitative study conducted at two out-patient gynecology clinics, Jones et al., 2011 interviewed 15 young women diagnosed with PCOS. The main objective of this study was to explore health-related quality of life among adolescents with polycystic ovary syndrome (PCOS).  Most participants discussed the prospect of infertility. Other concerns included exacerbation of symptoms, including hirsutism and weight gain.  A few adolescents reported experiencing pain (including frequent headaches, pelvic and abdominal pain) and tiredness, which they felt were related to their PCOS or associated treatments and negatively affected their physical functioning.  The adolescents felt that it was the main symptoms associated with the condition that had the most negative impact on quality of life, that is, weight, acne, hirsutism, infertility, and menstrual problems.  Eight of the participants reported varying degrees of hirsutism, describing excess hair growth as “horrible,” “annoying,” and “irritating.” Locations affected included the cheeks, upper lip, chin, neck, chest, arms, lower back, posterior thigh, bikini line, feet, and lower abdomen.  Irregular and absent periods caused great emotional distress amongst participants, particularly due to the prospect of infertility. Participants described feeling “depressed,” “disappointed,” “embarrassed,” “different,” “upset,” “frustrated,” “not normal,” and “distressed.” The adolescents described the prospect of infertility as “upsetting,” “worrying,” and “scary.” Participants also reported feeling “low,” “gutted,” “concerned,” “depressed,” and “panic” when considering fertility. |
| Kitzinger & Willmott (2002) | Kitzinger C, Willmott J. ‘The thief of womanhood’: women’s experience of  polycystic ovarian syndrome. Soc Sci Med. 2002; 54(3):349-61. | In this study, interviews were conducted with 30 women with PCOS to explore their experience with the syndrome. Findings suggest that the women questioned their ability to be defined as ‘normal women’, two-thirds said that they felt ‘different’, or ‘abnormal’ and nine women specifically used the term ‘freaks’ to describe their experience of themselves. These feelings were mainly associated with the three core themes of excessive body and facial hair, irregular menstruation,and infertility. |
| Li et al. (2011) | Li Y, Li Y, Yu Ng EH, Stener-Victorin E, Hou L, Wu T, Han F, Wu X. Polycystic ovary syndrome is associated with negatively variable impacts on domains of health-related quality of life: evidence from a meta-analysis. Fertil Steril. 2011; 96(2):452-8. | Li et al., 2011 systematically reviewed the literature to identify the impact of polycystic ovary syndrome (PCOS) on specific health-related quality of life domains. In the meta-analysis, Li et al., 2011 reviewed the outcomes of 423 patients and 285 controls from 5 articles that used the Short Form 36 (SF-36) questionnaire.  Compared with controls, women with PCOS had lower scores in all SF-36 dimensions: including physical function, physical role function, bodily pain, general health, vitality, social function, emotional role function, and mental health.  They concluded that women with PCOS score lower in each dimension of the SF-36, mostly in the emotional role function. |
| Percy et al. (2009) | Percy CA, Gibbs T, Potter L, Boardman S. Nurse-led peer support group: experiences of women with polycystic ovary syndrome. J Adv Nurs. 2009; 65(10):2046-55. | Percy et al., 2009 explored the experiences of women with polycystic ovary syndrome attending a nurse-led support group. Qualitative interviews were conducted with 13 female patients in 2006. The participants attended a support group at a public hospital in the United Kingdom.  Patients described the atmosphere of the group as very positive and supportive in talking about their symptoms: “People aren’t reluctant to talk about their symptoms or what they have, and I would imagine that most people in their general life don’t go around saying, ‘I’ve got a terrible facial hair problem’, whereas in that room they feel that they’re able to talk about that.” |
| Tasali et al. (2006) | Tasali E, Van Cauter E, Ehrmann DA. Relationships between sleep disordered  breathing and glucose metabolism in polycystic ovary syndrome. J Clin Endocrinol  Metab.2006; 91(1):36-42. | Tasali et al., 2006 sought to determine the relationships between risk and severity of obstructive sleep apnea (OSA) and glucose metabolism in PCOS.  Cohort 1 included 40 nondiabetic women with PCOS who completed the Epworth Sleepiness Scale, the Pittsburgh Sleep Quality, and the Berlin Questionnaire to assess risk of OSA; 32 of the 40 women had an oral glucose tolerance test. Cohort 2 included eight women who had a sleep study, glycosylated hemoglobin level, and an oral glucose tolerance test. In cohort 1, 62.5% of the women had poor sleep quality by Pittsburgh Sleep Quality Index, and 18 (45%) had chronic daytime sleepiness by Epworth Sleepiness Scale. Thirty of the 40 women had a high risk of OSA by the Berlin Questionnaire. Women with high OSA risk had higher fasting insulin levels and homeostasis model assessment index compared with those with low OSA risk.  Among women with normal glucose tolerance, insulin levels were significantly higher in those at high *vs.* low OSA risk, independently of body mass index. Women in cohort 2 had rapid eye movement (REM)-predominant OSA with lower sleep efficiency, longer sleep latency, and less REM sleep than controls. Glycosylated  hemoglobin levels and the area under the glucose curve positively correlated with the apnea-hypopnea index and the number of oxygen desaturations in REM sleep.  Tasali et al., 2006 concluded that PCOS is associated with poor sleep quality, daytime sleepiness, and increased risk for OSA. Insulin levels and measures of glucose tolerance in PCOS are strongly correlated with the risk and severity of OSA. |
| Teede et al. (2010) | Teede H, Deeks A, Moran L. Polycystic ovary syndrome: a complex condition with  psychological, reproductive and metabolic manifestations that impacts on health  across the lifespan. BMC Med. 2010 30;8:41. | In this review, Teede et al., 2010 discussed the psychological and cardiometabolic features of PCOS. They noted that PCOS is a common complex condition in women associated with psychological, reproductive, and metabolic features. Both hyperandrogenism and insulin resistance contribute to pathophysiology of PCOS. Insulin resistance occurs in the majority of women with PCOS, especially those who are overweight, and these women have a high risk of metabolic syndrome, prediabetes, and type 2 diabetes. Addressing hyperandrogenism is clinically important and monitoring for and managing longer-term metabolic complications, including dyslipidaemia, impaired glucose intolerance, type 2 diabetes, and cardiovascular risk factors, is crucial. |
| The Rotterdam ESHRE/ASRM-sponsored PCOS consensus workshop group, (2004). | Rotterdam ESHRE/ASRM-Sponsored PCOS consensus workshop group. Revised 2003  consensus on diagnostic criteria and long-term health risks related to polycystic ovary syndrome (PCOS). Hum Reprod. 2004; 19 (1):41-7. | The 2003 Rotterdam consensus workshop concluded that PCOS is a syndrome of ovarian dysfunction along with the cardinal features hyperandrogenism and polycystic ovary (PCO) morphology. PCOS remains a syndrome. Therefore, no single diagnostic criterion (such as hyperandrogenism or PCO) is sufficient for clinical diagnosis. Its clinical manifestations may include: menstrual irregularities, signs of androgen excess, and obesity. Insulin resistance and elevated serum LH levels are also common features in PCOS.  Revised 2003 Diagnostic criteria include (2 out of 3):   1. Oligo- and/or anovulation 2. Clinical and/or biochemical signs of hyperandrogenism 3. Polycystic ovaries and exclusion of other aetiologies (congenital adrenal hyperplasias, androgen-secreting tumours, Cushing's syndrome) |
| Weiss & Bullmer (2011) | Weiss TR, Bulmer SM. Young women's experiences living with polycystic ovary syndrome. J Obstet Gynecol Neonatal Nurs. 2011 40(6):709-18. | Weiss and Bullmer, 2011 conducted a study to explore the psychosocial effects of living with polycystic ovary syndrome (PCOS) through the experiences of young women diagnosed with this endocrine disorder. Twelve young women with PCOS age 18 to 23 years were interviewed.  Patients reported problems with anovulation: “I would say the thing that bothered me the most was probably just feeling like not feminine . . . that I’d basically never ovulate, and I’m not fertile . . . it made me kind of jealous of other women . . .”  Others reported concerns with appearance:  “…I get really uneasy and I can lose my confidence when I think about my physical appearance. Just because I do have more facial hair than most people . . . and the weight gain—losing weight is really tough…”  Participants reported either struggling to maintain their weight or had already gained weight and expressed that they needed to get it under control. “I feel kind of gross. I don’t know why . . . I feel when I eat something. . . I’m gonna gain weight really quickly . . . I worry that I’m not going to be able to change and have problems.”  Even with regular, strenuous exercise some women had trouble shedding pounds. Participants relayed frustration with not being able to lose weight even though being active, “I exercise all the time . . . and it doesn’t really come off very easily. It shouldn’t be like that since I’m only 19.”  Additionally, participants discussed how they handled hirsutism, like regularly undergoing costly laser hair removal treatment. |
| Womenshealth.gov | http://www.womenshealth.gov/ Accessed 7/31/2013 at 10:32 AM EST. | Womenshealth.gov, the website for the Office on Women’s Health, U.S. Department of Health and Human Services, has a PCOS fact sheet which lists some of the symptoms of PCOS. These include:   - Infertility because of not ovulating. In fact, PCOS is the most common cause of female infertility. - Infrequent, absent, and/or irregular menstrual periods - Hirsutism or increased hair growth on the face, chest, stomach, back, thumbs, or toes - Cysts on the ovaries - Acne, oily skin, or dandruff - Weight gain or obesity, usually with extra weight around the waist - Male-pattern baldness or thinning hair - Patches of skin on the neck, arms, breasts, or thighs that are thick and dark brown or black - Skin tags or excess flaps of skin in the armpits or neck area - Pelvic pain - Anxiety or depression - Sleep apnea |
