# Supplementary figures and images for: Understanding polycystic ovary syndrome from the patient perspective: a concept elicitation patient interview study
Source: Health Qual Life Outcomes. 2017 Aug 18;15:162. doi: 10.1186/s12955-017-0736-3 (PMC5562990; doi:10.1186/s12955-017-0736-3)

**Supplementary Figure –** Draft PCOS disease model based on literature analysis


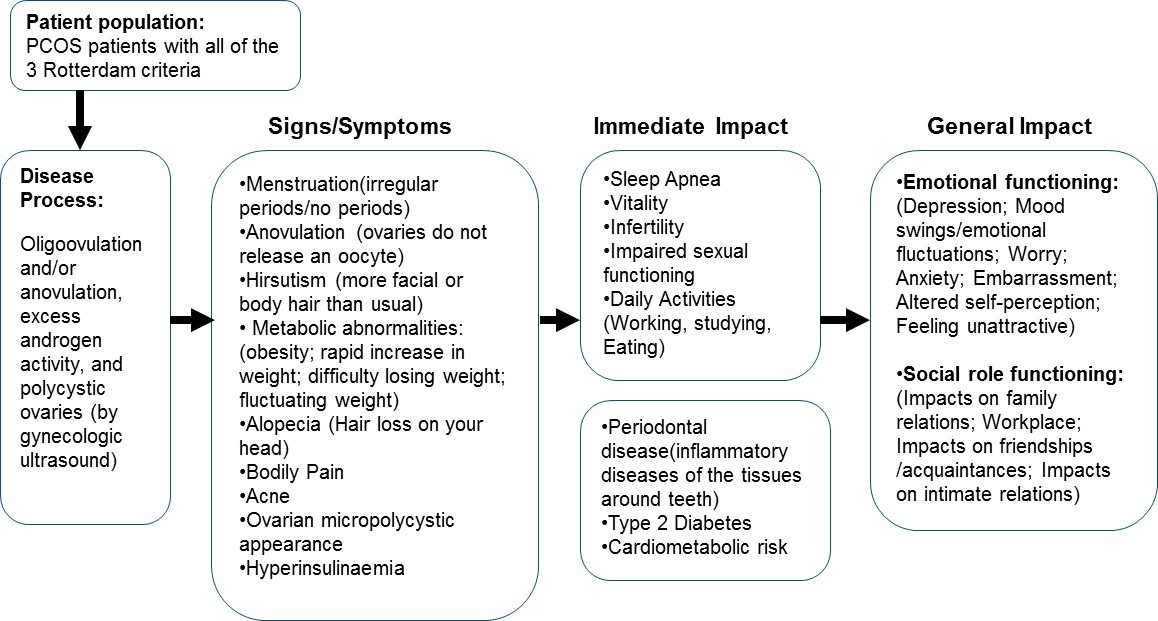

Supplement: Supplementary file 3 — Draft PCOS disease model. (DOCX 182 kb) [file 12955_2017_736_MOESM3_ESM.docx]
